# Navigating Gene Therapy Access: The Case of Bulgaria in the Context of the EU Regulatory Landscape

**DOI:** 10.3390/healthcare12040458

**Published:** 2024-02-11

**Authors:** Kostadin Kostadinov, Yuliyana Marinova, Kostadin Dimitrov, Eleonora Hristova-Atanasova, Georgi Iskrov, Rumen Stefanov

**Affiliations:** 1 Department of Social Medicine and Public Health, Faculty of Public Health, Medical University of Plovdiv, 4002 Plovdiv, Bulgaria; yuliyana.marinova@mu-plovdiv.bg (Y.M.); kostadin.dimitrov@mu-plovdiv.bg (K.D.); eleonora.hristova@mu-plovdiv.bg (E.H.-A.); georgi.iskrov@mu-plovdiv.bg (G.I.); rumen.stefanov@mu-plovdiv.bg (R.S.); 2 Institute for Rare Diseases, 4023 Plovdiv, Bulgaria

**Keywords:** gene therapy, access, pricing, reimbursement, Bulgaria

## Abstract

Gene therapies (GTs) have recently emerged as revolutionary personalized therapeutic options. Despite their promising potential, challenges such as uncertainty regarding long-term health benefits and safety, along with extreme price tags, pose significant obstacles to patient access. Within the EU, the European Medicines Agency plays a pivotal role with regards to GT market authorization. However, national authorities are responsible for pricing and reimbursement, which results in fragment patient access within the EU. This study aimed to provide an overview of the complex landscape of post-market authorization accessibility for GT products in Bulgaria, comparing it with neighboring EU countries. We applied a mixed-methods approach, including desk research, public data requests, and list price comparisons. As of 1 April 2023, 14 GTs had a valid market authorization at the EU level. In Bulgaria, Kymriah^®^ was the only GT included in the Positive Drug List (PDL), with an official list price of EUR 335,636.94. Similar results were found in Romania, whereas five GTs were included in Greece’s PDL. Additionally, Zolgensma^®^ was found accessible in Bulgaria through an alternative individual access scheme at an estimated price of EUR 1,945,000.00. In conclusion, this study emphasized targeted policy interventions to address health inequalities and to ensure timely access to GTs within the EU.

## 1. Introduction

### 1.1. Aim of the Study

This study aimed to assess the current level of accessibility of gene therapy medicinal products (GTs) in Bulgaria, with a specific emphasis on the different legal tools to access GTs and their budgetary impact, respectively. Access to GTs in Bulgaria is further compared to those in neighboring European Union (EU) member states—Greece and Romania. Finally, key factors influencing access to GTs and the main challenges for this process in Southeastern Europe are outlined and explored.

### 1.2. Background of the Problem

Over the past decade, GTs have become a groundbreaking approach, marking a paradigm shift in the pursuit of personalized healthcare solutions [1,2,3]. According to the official definition in the EU, a GT involves the introduction of recombinant nucleic acids into the human body with the specific aim of regulating, repairing, replacing, adding, or deleting specific genetic sequences [4]. GTs are categorized under the group of advanced therapy medicinal products (ATMPs) [5]. Most of the ATMPs are currently in the experimental stage, undergoing clinical trials to assess their safety and effectiveness. Nevertheless, the positive clinical results achieved with already-approved ATMPs drive further research developments and attract substantial investments [6]. Despite these achievements, clinical uncertainty regarding long-term benefits and potential adverse effects, combined with an extreme price tag, presents unique challenges to patient access [7,8,9].

### 1.3. EU Regulatory Framework

The EU’s ATMP legislation is centralized, signifying collaborative efforts among various stakeholders. The Committee for Advanced Therapies (CAT) within the European Medicines Agency (EMA) plays a crucial role in the market authorization process, evaluating safety and efficacy. The CAT’s evaluations impact the Committee for Medicinal Products for Human Use (CHMP) recommendations and, thereby, influence decisions made by the EMA. In addition, the CAT offers guidance on ATMP classification, provides scientific advice covering clinical trial designs and endpoint assessments, and contributes to efficacy follow-up and risk management procedures [10].

Regulatory mechanisms like accelerated assessment and conditional marketing authorization are often applied to ATMPs. Over half of authorized GTs underwent evaluation under the PRIME (PRIority Medicines) scheme, intensifying support for medicines targeting unmet health needs. PRIME facilitates support from the EMA and encourages early engagement with stakeholders in a submission-readiness meeting a year before the market authorization submission. As part of the PRIME benefits, a scientific coordinator is appointed, and a fee exemption for scientific advice is provided to applicants from the European Economic Area [11]. Notably, early access tools are not mutually exclusive. PRIME-designated medicines often combine accelerated assessment, compassionate use, or orphan designation [12]. Additionally, the EMA provides various financial and regulatory incentives, such as fee reductions and waivers tailored specifically for small and medium enterprises and academic institutions [13].

Despite their central role in regulating and approving GT products, the EU bodies do not influence post-authorization pricing or reimbursement decisions at the national level. These decisions are decentralized, considering the healthcare system characteristics and patient needs of each member state. Regional or national regulators and payers typically oversee access negotiations and make decisions about potential reimbursement strategies and distribution plans [14]. Health technology assessment (HTA), which takes into account factors like cost-effectiveness and budgetary impact, is frequently used to guide this process [15].

### 1.4. Bulgarian Pricing and Reimbursement Process

Patient access to GTs within the general reimbursement scheme in Bulgaria entails a complex procedure based on several components (Table 1). The Medicinal Products in Human Medicine Act (MPHMA) [16] sets the overall regulatory framework in the country. Key stakeholders in the general reimbursement process are the Ministry of Health (MoH), the National Council on Prices and Reimbursement of Medicinal Products (NCPRMP), and the National Health Insurance Fund (NHIF).

At the beginning, market authorization holders apply to the NCPRMP for pricing, HTA, and inclusion in the Positive Drug List (PDL). These three procedures are usually initiated at the same time. To be included in the PDL (and thus be eligible for reimbursement), an innovative therapy must already be covered by public funds in at least 5 out of 17 reference EU countries. For orphan-designated products, this list extends to all EU member states [17].

Pricing and HTA procedures are mandatory for all original medicinal products. NCPRMP applies external reference pricing based on the lowest product price from a legally defined list of ten EU countries. The final maximum price is computed by incorporating fixed margins for wholesalers and retailers, along with a 20% value-added tax. The official list price is subject to monthly updates based on the corresponding changes in the lowest price from the reference countries [17].

The NCPRMP also acts as a national HTA body in Bulgaria. Legally, the HTA stage requires additional evidence from HTA reports from the UK, France, Germany, and Sweden. The incremental cost-effectiveness ratio threshold for value efficiency is set at three times GDP per capita [17].

The NHIF, being the single public healthcare payer, is responsible for the final stage of the reimbursement decision-making process. This step involves yearly reimbursement negotiations and agreements, which require a minimum 10% discount on the official list price. However, these agreements are confidential, and the exact discount rates are not publicly available. The NHIF also determines a percentage of coverage, along with additional patient eligibility criteria and outcome effectiveness monitoring if needed. Once a therapy is included in PDL, the NCPRMP conducts mandatory reviews of its reimbursement status every three years. Additionally, the NCPRMP has the authority to initiate a HTA reassessment procedure. This regulatory approach aims to ensure ongoing evaluation and alignment with evolving healthcare considerations [18].

### 1.5. Bulgarian Induvial Access Schemes

Besides the general reimbursement scheme, patient access to GTs can also be obtained through two options, via individual schemes. Both are used in severe cases where no other therapeutic alternatives are available, and the currently applied standards of care do not result in clinical improvement (Table 1).

The first strategy, which is subject to MoH Ordinance 10 of 2011, operates on a case-by-case basis. The administrative process involves a comprehensive review by an ad hoc commission consisting of three doctors, a pharmacist, and a lawyer. This scheme enables the administration of medicinal products not listed in the PDL, non-authorized treatments, or those included in the PDL but applied in indications not included in the product characteristics (off-label use). Off-label approval is granted when available data support potential clinical benefits or when the proposed innovative treatment has been applied in similar cases in other countries. Typically, the funding for off-label treatments comes from the state budget. However, this regulation also allows pharmaceutical companies to finance products not listed in the PDL through specially designed compassionate use programs [19].

MoH Ordinance 2 of 2019 constitutes the second individual scheme in Bulgaria. This scheme enables patients under 18 years of age to access specialized medical care beyond the coverage of mandatory health insurance. The scope of this procedure includes rare diseases, cancer, and congenital hematological diseases. When that strategy is used, the case applications are assessed by an ad hoc commission, comprising a representative of the executive body of NHIF and medical experts in the relevant field. Upon approval, the price of the innovative product is negotiated directly between NHIF and the manufacturer. The NHIF covers the full payment, which is subsequently compensated by a direct transfer from the state budget [20].

## 2. Materials and Methods

The study applied a mixed-methods approach, combining the following steps: desk research, public data requests, and cost comparison. Data collection took place between 27 March 2023 and 10 April 2023.

### 2.1. Desk Research

In the initial phase of the analysis, a comprehensive list of all the EMA GTs with a valid market authorization as of 26 March 2023 was compiled. The search strategy incorporated key terms, including “gene therapy(ies)”, “gene product(s)”, “gene medicinal product(s)”, “advanced therapy medicinal product(s)”, and “ATMP(s).” Relevant information was systematically retrieved through searches on the EMA’s website, the European Public Assessment Reports (EPARs) database, and the EU’s Register of Medicinal Products for Human Use.

Subsequently, each individual medicinal product underwent scrutiny in the electronic database of the Bulgarian PDL to determine its official list price and reimbursement status. Unlike traditional static lists, the Bulgarian PDL operates in an electronic open data format (https://portal.ncpr.bg/registers/pages/register/list-medicament.xhtml, accessed on 1 April 2023), providing a dynamic and accessible platform for stakeholders in the healthcare system. The PDL database is updated monthly. Our search utilized both the market name of the medicinal product and the international nonproprietary name (INN).

Additionally, a search of databases containing HTA reports, prepared by the NCPRMP, was conducted on 1 April 2023, to ascertain if any assessments had been conducted for the identified GTs.

### 2.2. Data Requests

On 27 March 2023, formal public data requests were dispatched to both the NHIF and the NCPRMP. The NHIF request sought information on payments from public funds for GT treatments under the national framework contract (a general reimbursement scheme) or the mechanisms framed under Ordinances 2 and 10 (an individual access scheme), providing real-time insights into the budgetary landscape for GTs within the national healthcare system. Simultaneously, the NCPRMP request was made to gain a thorough understanding of the regulatory aspects related to GTs, specifically accessing public information regarding the initiated procedures of HTAs and/or the registration of an official price of GT medicinal products in Bulgaria.

### 2.3. Comparison of Official List Prices

For the identified GTs that are currently accessible in Bulgaria (either by a general reimbursement scheme or an individual access scheme), we compared the official list prices to those in neighboring EU countries—Greece and Romania. This selection was deliberate, considering geographical proximity, epidemiological, economic, and sociocultural similarities, and comparable healthcare systems [21]. The official PDL databases [22,23,24] were screened to acquire up-to-date pricing information. The official prices in Bulgaria were converted in EUR using the fixed rate of the current currency board. The official prices in Romania were converted in EUR using the exchange rate, as set by the Romanian National Bank on 1 April 2023 [25]. The analysis did not consider the not-publicly available discount agreements between the manufacturers and the payer, which are similarly required by the corresponding Greek and Romanian legislation [26,27,28].

### 2.4. Analysis

The data analysis was conducted utilizing R version 4.3.2. Data visualization was performed using the ggplot2 package. The results were summarized using descriptive statistics. Categorical variables were summarized with counts and percentages. Differences in proportions were assessed using the chi-squared test. The significance level was set at 0.05. Mean cost per treatment was calculated following the EMA-approved summary of product characteristics, using identical dosage assumptions to ensure cost comparability. Only the direct GTs costs were considered. Other remaining expenditures, such as costs for diagnostics, consultations, and hospitalizations were not included. The budgetary impact for Bulgaria was estimated as a proportion of the National Health Insurance Fund Medicines Expenditure (NHIF ME) according to the NHIF’s budgets for 2021 and 2022.

## 3. Results

### 3.1. Market Authorization of Gene Therapies in the EU

**Figure 1:**
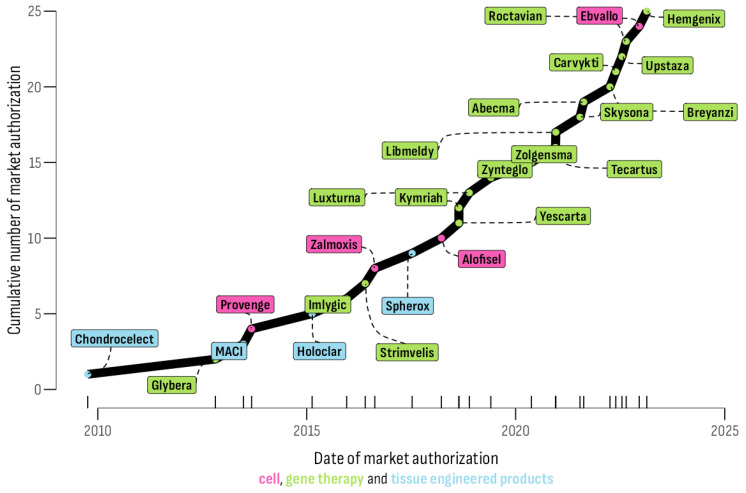
Time distribution of gene therapies garnering positive market authorization.

A total of 25 ATMPs that have received positive market authorization via a centralized EU procedure were identified. A significant difference among the types of ATMP was observed (χ^2^ = 13.52, df = 2, *p* < 0.05). GTs accounted for the majority (n = 17; 68%), followed by cell therapies (CTs) (n = 4; 16%), and tissue-engineered products (TEPs) (n = 4; 16%) (Figure 1).

Orphan designation varied significantly among the types of ATMPs (χ^2^ = 7.1014, *p* = 0.0287). The highest rate was found for GTs (n = 15/17, 88.2%), followed by CTs (n = 3/4, 75%), and TEPs (n = 1/4, 25%). The PRIME designation was also more commonly applied to GTs (n = 10/17, 58.8%) compared to CTs (n = 1/4, 25%), while none of the TEPs were approved under the PRIME scheme. Only one GT product (Breyanzi^®^) received marketing authorization exclusively under PRIME. Additionally, seven GT products benefited from both PRIME and orphan designation. Imlygic^®^ was the only authorized GT that did not use any of the available incentives.

Withdrawals were observed for two GTs (Skysona^®^ and Zynteglo^®^), one CT (Zalmoxis^®^), and one TEP (MACI^®^). In addition, the first approved GT product in the EU, Glybera^®^, authorized in 2012, was withdrawn in 2018 following the marketing-authorization holder’s decision not to apply for a renewal.

Excluding withdrawn products, the final list consisted of 14 GT products with a valid market authorization by the time of the study, with 12 (84%) tailored specifically for rare diseases (Table 2).

### 3.2. Gene Therapies in the Bulgarian Positive Drug List

Only one GT (Kymriah^®^) underwent a pricing procedure with a NCPRMP decision on 2 July 2020. The official list price for the product was not changed from the pricing procedure to the research date and was EUR 335,636.94 per package (and subsequently, this was also the cost per patient since one package covers the whole treatment course). However, Kymriah^®^ is currently not included on the PDL and therefore is not eligible for reimbursement by the NHIF. For all the remaining 13 GTs, we could not find any evidence of pricing or inclusion in the PDL, leading us to the conclusion that the market authorization holder did not start any such procedures in Bulgaria.

### 3.3. HTA Reports for Gene Therapies

The Bulgarian HTA agency’s databases (NCPRMP) include publicly available information on positive and negative HTA reports dating back to 2019. However, a comprehensive search revealed that none of the 14 GTs have completed this process. In response to a public data request, the official answer from NCPRMP stated that the agency initiated both HTA assessment and pricing for Zolgensma^®^ on 28 September 2021. However, no final decisions are publicly available yet.

### 3.4. Alternative Funding Strategies and Budjetary Impact

The NHIF data obtained through a public request revealed that four patients were treated with Zolgensma^®^ under the framework of the MoH Ordinance 2 of 2019, with a total cost of EUR 11,402,550.16, or EUR 2,850,637.54 per patient. The direct medicinal expenditures were EUR 5,601,942.61 for two patients treated in 2021 and EUR 5,800,607.55 for two patients treated in 2022. These four payments accounted for 0.8% and 0.7% of all NHIF budgets allocated for medicinal treatment in the respective years. There was no available data for the remaining 13 GTs, implying that they have not been funded through the NHIF or the state budget (under either the MoH Ordinance 2 or MoH Ordinance 10).

### 3.5. Official List Prices

The pricing and reimbursement status of the identified 14 GTs with a valid market authorization were searched and checked in the PDL databases of Greece and Romania. Five out of the fourteen GTs (Luxturna^®^, Zolgensma^®^, Yescarta^®^, Tecartus^®^, and Kymriah^®^) were included in Greece’s PDL, resulting in a 36% inclusion rate, while only one (Kymriah^®^) was found in Romania’s PDL (7%) (Table 1). Comparison was made between the list price for Zolgensma^®^ in Greece and the negotiated price disclosed by the NHIF as well as the list price for Kymriah^®^ in both Greece and Romania.

Up to 1 April 2023, Zolgensma’s list price in Greece was EUR 2,134,478 per dose. A direct comparison resulted in an absolute difference of EUR 716,159, with the product’s price being higher in Bulgaria. This difference is remarkable due to the fact that the treatment costs in Greece could be expected to be even lower considering the applicable discount agreements. Moreover, Bulgarian expenditures might be inflated due to direct market negotiations under the MoH Ordinance 2 of 2019 and the lack of formal pricing procedure. For Kymriah^®^, a direct comparison indicated a Bulgarian list price 1.05 times higher than Greece, with an absolute difference of EUR 16,270.17. Compared to Romania, the estimated absolute difference was EUR 3029.37.

## 4. Discussion

Our study identified a smaller number of accessible GTs in Bulgaria in comparison to Greece and Romania. Furthermore, the implementation of tools for individual patient access to GTs in Bulgaria seemed to result in elevated purchase expenses. Nevertheless, our research is limited, and no overall conclusions about the uptake of GTs and ATMPs should be drawn.

On the other hand, the main health policy question here should not be about the uptake of these innovative therapies but rather the availability of legal mechanisms for patients to access this kind of treatment [28]. Patients, regardless of their diagnosis, should be able to obtain timely, adequate, and effective therapy. The lack of therapies or implementation of access schemes that are not optimal exacerbates health inequalities and leads to excess costs.

### 4.1. Balancing Cost, Access, and Ethical Responsibilities

GTs deviate significantly from traditional medicinal products, exhibiting unique characteristics in development, production, therapeutic mechanism, and clinical benefits [1,29]. As a form of personalized medicine, large-scale production is often impractical, justifying their elevated costs [2]. This uniqueness not only adds complexity but, when combined with inherent long-term clinical uncertainties, makes patient access to GTs highly problematic [30,31]. On one hand, denying access to these pioneering treatments not only poses a risk for patients but also raises ethical concerns [32], emphasizing the moral obligation to ensure fair access, especially in cases where GTs are the only therapeutic option in place [33]. On the other hand, manufacturers grapple with delicately balancing shareholder interests and societal responsibilities. As a convincing return on investment becomes pivotal for sustainable development [34,35], often a paradigm shift in pricing and reimbursement models is needed [36].

### 4.2. Market Authorization and Reimbursement Challenges

Two critical points stand out in the complex journey of patient access to GTs: market authorization and reimbursement decision-making. Market authorization serves as the regulatory stamp of approval, confirming the safety and efficacy of these therapies [37]. The small number of participants in GT clinical trials, however, can pose a challenge to this process and raise questions about the generalizability of the results [8]. The growing number of individuals turning to compassionate use programs compels regulators to base extrapolated safety data on N-of-1 study designs, introducing a dynamic shift from traditionally approved randomized trials [6,38]. Further methodological issues, including the utilization of single-arm trials and historical cohorts, as well as inappropriate comparators, additionally induce uncertainty about the effectiveness and durability of therapeutic effects [7,39].

To overcome these hurdles, the EMA has implemented strategic measures like the PRIME and Orphan Designation programs [11,40,41]. The CAT and scientific adversity communities add specialized expertise, helping to facilitate the evaluation of advanced therapeutic medicinal products. Financial incentives also streamline the regulatory process, facilitating innovative treatment development [11]. This collaborative effort has yielded tangible results, as explored in the current research with 25 market-authorized ATMPs. Notably, the majority of them (68%) are GTs, with 88% receiving an orphan designation and 58% approved under the PRIME scheme. Nevertheless, the EU does not hold the forefront in GT development, as China and the United States have a higher number of market-approved GTs [42,43].

The second challenge to achieving GT access lies in reimbursement decision-making, a decentralized process within the EU that fragments the common market and leads to significant health inequalities [7,44]. Our study revealed that in Bulgaria, only one GT product, Kymriah^®^, has undergone a pricing procedure, but it is not yet included in the PDL, rendering it ineligible for public funds reimbursement. A second GT (Zolgensma^®^) has initiated pricing and HTA procedures, but official decisions are still pending, despite this process starting over a year ago [45]. Even among neighboring countries that are comparable in terms of health systems and health resources, such as Bulgaria and Greece [46], notable discrepancies in these reimbursement decisions are observed. For instance, in Greece, five out of fourteen GTs are listed on the PDL, indicating a 36% inclusion rate. The official list price of Zolgensma^®^ is 1.48 times lower in Greece than in Bulgaria, a finding that persists even when potential discount agreements are not considered. This emphasizes the intricate nature of reimbursement decisions and highlights the need for a more transparent approach to ensure equitable access to GTs across diverse healthcare systems [47,48].

### 4.3. Innovative Reimbursement Models

Typically, reimbursement decisions are based on the HTA results [15]. However, several studies emphasize that traditional HTA methods cannot quantify the overall patient benefits provided by GT [49]. Furthermore, substantial heterogeneity exists in the clinical indications of GTs [50]. While some are categorized as life-saving medications, often intended for children, the majority of these products are formulated to address rare cancers in adults [2,51]. In those cases, the scarcity of survival data poses a significant challenge in determining the appropriate time frame for benefit, particularly given the heightened sensitivity of cost-effectiveness analysis to the applied discount rates [30,42,50].

Reimbursement models that involve risk-sharing, such as pay-for-performance arrangements and outcome-based rebates, offer innovative solutions to traditional challenges in reimbursement [52]. These models, like product performance agreements that link payments to clinical outcomes, effectively distribute the financial responsibility associated with high gene therapy prices. They promote a balanced risk-sharing approach between payers and manufacturers. This approach enhances patient access without significantly escalating budgetary impact [53]. Annuity payments, akin to risk-sharing agreements, offer reimbursements in annual installments based on specific clinical results [54]. Payments linked to therapeutic outcomes (an alternative model), involves payers compensating manufacturers upon achieving pre-negotiated treatment results. These strategies are meant to reduce budgetary impact and uncertainty, as well as to stimulate GT development and access [53]. These goals suggest why there is a need to refine value assessment frameworks from a broader societal perspective. Such steps have been taken by several EU member states not only for GTs like Yescarta^®^ and Kymriah^®^ [55,56,57,58] but also for other personalized medicine interventions, such as genomic testing [59].

However, these innovative reimbursement models are not without limitations. The administrative burden due to lack of unified implementation strategies can be substantial [60]. Risk-sharing models can be particularly challenging, as they require additional resources for monitoring and data collection [61]. Often these models lead to increased transaction costs, which can be a significant barrier for SMEs and academic institutions. Furthermore, the lack of transparency and the potential for conflicts of interest can undermine the credibility and increase health inequalities [62].

### 4.4. Limitations

There are several limitations to our study’s findings. First, we only focused on public fund reimbursement and did not explore any options for private funding of GT access. This is reasonable because of the extremely high official prices of GTs. Except for GT treatment provided within clinical trials, it would be very unlikely for patients to access GTs outside the scope of public funds.

Second, we compared the official list prices of GTs. Those prices, however, are not the ones that are actually used for reimbursement decisions. Instead, regulators and payers nowadays require certain discounts and rebates. The latter are confidential and therefore not publicly available. The latter restriction ultimately makes international comparisons very difficult.

Third, while we could request public data about alternative individual access schemes in Bulgaria, we were not able to do the same regarding Greece and Romania. It is realistic to expect that similar access mechanisms exist and are applied in these jurisdictions as well.

Fourth, we analyzed GT access in three neighboring EU member states. Our sample was limited, and any extrapolations from this research must be cautious. Nevertheless, our findings suggest very limited access to GTs in Southeastern Europe, thus ringing the bell for targeted policy measures to address these significant health inequalities.

Finally, we explored whether and how GTs and ATMPs benefit from the various incentives that the EU applies to foster the research and development of these innovative health technologies. There is a lot of variability between and within the three main types of ATMPs. Furthermore, the sample size was relatively small. Therefore, no general conclusions could be drawn about the pattern of use of those legal instruments and their impact on the progress of ATMPs.

## 5. Conclusions

Bulgaria is currently applying a combination of general reimbursement and individual schemes to provide access to GTs. While, on paper, these tools should secure a GT-friendly regulatory environment, we found a lower scope in terms of the number of accessible treatments compared to neighboring Greece and Romania. Additionally, individual access schemes in Bulgaria were associated with higher purchasing costs. This is a particularly troublesome situation considering the country’s limited resources.

We call for policy actions at both the EU and national levels. We recommend exploring EU-wide incentives for market authorization holders and manufacturers to undergo national pricing procedures in all the EU member states. At the national level, we suggest considering novel financial models, including annuity payments, in order to mitigate the budgetary risks that are associated with GTs. Addressing all these challenges is vital to establishing a more uniform and equitable landscape for GT access within the EU, fostering a supportive environment for patients and their families, and encouraging further innovations in this transformative field of medicine.

## Figures and Tables

**Table 1:** Comparison of gene therapy access schemes in Bulgaria.

Scheme Framework Component	General Scheme	Individual Scheme Ordinance 10	Individual Scheme Ordinance 2
Regulator	MoH, NCPRMP, NHIF	MoH, BDA Ad hoc commission (*three doctors, a pharmacist, and a lawyer*)	MoH, NHIF Ad hoc commission (*a representative of the executive body of NHIF and medical experts*)
Scope	General reimbursement	Off-label Compassionate Use	Age limitation (*applicable only to patients under 18 years*)
Reimbursement of non-authorized products	No	Yes	No
PDL inclusion procedure	Yes	No	No
Pricing procedure	Yes Mandatory external reference pricing (*Monthly update based on the lowest external reference price*)	No Direct negotiation process	No Direct negotiation process
HTA procedure	Yes (*Reappraisal every 3 years if deemed necessary*)	No	No
Discounts	Yes (*Mandatory confidential discounts of at least 10%*)	No	No
Funding source	NHIF (*Review of the reimbursement status and PDL inclusion every 3 years*)	State budget Market authorization holders (*if applicable*)	State budget transfer to NHIF

MoH—Ministry of Health; NCPRMP—National Council on Prices and Reimbursement of Medicinal Products; NHIF—National Health Insurance Fund; BDA—Bulgarian Drug Agency; PDL—Positive Drug List; HTA—Health Technology Assessment.

**Table 2:** Official list prices (in EUR) for gene therapy products authorized by the EMA up to 26 March 2023.

GT Product	Therapeutic Area	Date of MA	Orphan Designation	PRIME	Bulgaria	Greece	Romania
Imlygic^®^	Melanoma	16 December 2015	❌	❌	-	-	-
Strimvelis^®^	Severe Combined Immunodeficiency	26 May 2016	✅	❌	-	-	-
Yescarta^®^	Lymphoma	23 August 2018	✅	✅	-	321,818	
Kymriah^®^	Lymphoma/Leukemia-Lymphoma	22 August 2018	✅	✅	335,637	319,367	332,490
Luxturna^®^	Leber Congenital Amaurosis/ Retinitis Pigmentosa	22. November 2018	✅	❌	-	378,609	-
Zolgensma^®^	Muscular Atrophy, Spinal	18 May 2020	✅	✅	2,850,638 *	2,134,478	-
Tecartus^®^	Lymphoma, Mantle-Cell	14 December 2020	✅	✅	-	367,416	-
Libmeldy^®^	Leukodystrophy, Metachromatic	17 December 2020	✅	❌	-	-	-
Abecma^®^	Multiple Myeloma	18 August 2021	✅	✅	-	-	-
Breyanzi^®^	Lymphoma/ Mediastinal Neoplasms	4 April 2022	❌	✅	-	-	-
Carvykti^®^	Multiple Myeloma	25 May 2022	✅	✅	-	-	-
Upstaza^®^	Aromatic L-amino acid decarboxylase deficiency	18 July 2022	✅	❌	-	-	-
Roctavian^®^	Haemophilia A	24 August 2022	✅	❌	-	-	-
Hemgenix^®^	Hemophilia B	20 February 2023	✅	✅	-	-	-

* Estimated price in EUR based on official NHIF data.

## Data Availability

The complete data repository for this study is available on GitHub https://github.com/kostadinoff/Navigating-Gene-Therapy-Access--The-case-of-Bulgaria-in-the-context-of-EU-Regulatory-Landscape.git (created on 1 December 2023).
